# Insights from cardiovascular magnetic resonance imaging supporting the selfish brain hypothesis of arterial hypertension

**DOI:** 10.1186/1532-429X-17-S1-P405

**Published:** 2015-02-03

**Authors:** Jonathan C Rodrigues, Emma C Hart, Neelam Hassan, Mandy Williams, Amy E Burchell, Laura E Ratcliffe, Angus K Nightingale, Julian F Paton, Nathan E Manghat

**Affiliations:** 1 CMR Unit, NIHR Cardiovascular Biomedical Research Unit, Bristol Heart Institute, Bristol, UK; 2 School of Physiology and Pharmacology, The University of Bristol, Bristol, UK; 3 Cardionomics Research Group, Bristol Heart Institute, Bristol, UK; 4 Foundation School, Severn Postgraduate Deanery, Bristol, UK; 5 Department of Radiology, Bristol Royal Infirmary, Bristol, UK

## Background

Brainstem hypoperfusion may evoke systemic hypertension as a mechanism to boost cerebral blood flow: the selfish brain hypothesis. This notion is supported by evidence of vertebral artery hypoplasia (VAH) in hypertensive rat models. We determined the prevalence of anatomical variations of posterior cerebral circulation which could predispose to brainstem hypoperfusion in a hypertensive human cohort.

## Methods

**Figure 1:**
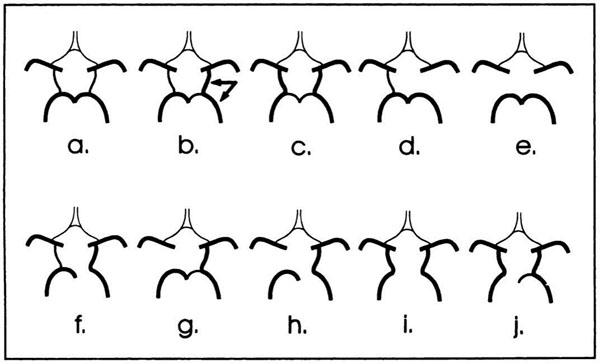
**Posterior Circle of Willis anatomical variations.** Adapted from: Krabbe-Hartkamp MJ et al. Circle of Willis: morphological variation on three-dimensional time-of-flight-MR angiograms. Radiology 1998; 207: 103-111.

The study was conducted in accordance with The Governance Arrangements for Research Ethics Committees. Patients referred from our tertiary hypertension clinic underwent comprehensive MR assessment including CMR and 3D time-of-flight MR angiography (MRA) imaging at 1.5T. MRAs were double reported by a blinded Neuroradiologist. VAH was defined as diameter <2mm, and compared to published prevalence VAH data in 306 healthy controls. Circle of Willis (CoW) morphology was classified according to previously published reference standards from 50 healthy subjects at 1.5T time-of-flight MRA (Figure 1). Demographic data, including office systolic (SBP) and diastolic blood pressures (DBP) and CMR-derived indexed left ventricular mass (LVM) were recorded. Continuous variables were compared using Student *t*-tests and categoric variables by Fisher exact test (*p*<0.05 = significant).

## Results

One hundred and twenty one (n=121) MRAs were studied (mean age 51.7±14.5 vs controls: 62.±10.3 years, p>0.05). VAH was more prevalent in hypertensives than controls [EH1] (51.2% vs 26.5%, p<0.0001), with right-sided VAH [69.4% (n=43/62)] most common. There was a higher prevalence of incomplete posterior CoW in hypertensives (62.0% vs 38.0%, p<0.01). Of the posterior CoW variants, complete disconnection of anterior/posterior CoW was more common in hypertensives (30.6% vs 10.1%, p<0.0001). Prevalence of both VAH and incomplete posterior CoW was higher in hypertensives compared to expected prevalence for controls (38.0% vs 10.1%, p<0.0001).

Amongst the resistant hypertension subgroup, VAH and incomplete CoW was more common than normal configuration [62.2%, n=23/37 vs 35.7%, n=30/84, p<0.01]. No difference in posterior circulation configuration prevalence was seen in difficult to treat (p=0.2497) or drug-intolerant (p=0.4218) hypertension. VAH and incomplete CoW was less common in young-onset hypertension [5.4% (n=2/37) vs 29.8% (n=25/84), p<0.005]. No differences in SBP (p=0.5757), DBP (p=0.8076) or indexed LVM (p=0.1553) were demonstrated between hypertensive patients with or without VAH and incomplete CoW.

## Conclusions

A significantly higher prevalence of posterior cerebral circulation anatomical variations, which could lead to increased vascular resistance, is demonstrated in hypertensives compared to controls. VAH and incomplete posterior CoW is most prevalent in resistant hypertensives. Our data support the selfish brain hypothesis of brainstem hypoperfusion as a driver of hypertension.

## Funding

NIHR Cardiovascular Biomedical Research Unit, Bristol Heart Institute

JCLR: Clinical Society of Bath Postgraduate Research Bursary

ECH: BHF grant IBSRF FS/11/1/2840.

